# Specific and label-free endogenous signature of dystrophic muscle by Synchrotron deep ultraviolet radiation

**DOI:** 10.1038/s41598-023-37762-1

**Published:** 2023-07-04

**Authors:** Laurence Dubreil, Noreddine Damane, Romain Fleurisson, Marine Charrier, Julien Pichon, Isabelle Leroux, Cindy Schleder, Mireille Ledevin, Thibaut Larcher, Frédéric Jamme, John Puentes, Karl Rouger

**Affiliations:** 1 Oniris, INRAE, PAnTher, 44300 Nantes, France; 2 IMT Atlantique, Lab-STICC, UMR CNRS 6285, 29238 Brest, France; 3 Synchrotron SOLEIL, BP48, L’Orme Des Merisiers, 91120 Gif-Sur-Yvette, France

**Keywords:** Wide-field fluorescence microscopy, Fluorescence spectroscopy, Muscle stem cells, Skeletal muscle, Stem-cell research, Preclinical research, Pathogenesis

## Abstract

Dystrophic muscle is characterized by necrosis/regeneration cycles, inflammation, and fibro-adipogenic development. Conventional histological stainings provide essential topographical data of this remodeling but may be limited to discriminate closely related pathophysiological contexts. They fail to mention microarchitecture changes linked to the nature and spatial distribution of tissue compartment components. We investigated whether label-free tissue autofluorescence revealed by Synchrotron deep ultraviolet (DUV) radiation could serve as an additional tool for monitoring dystrophic muscle remodeling. Using widefield microscopy with specific emission fluorescence filters and microspectroscopy defined by high spectral resolution, we analyzed samples from healthy dogs and two groups of dystrophic dogs: naïve (severely affected) and MuStem cell-transplanted (clinically stabilized) animals. Multivariate statistical analysis and machine learning approaches demonstrated that autofluorescence emitted at 420–480 nm by the *Biceps femoris* muscle effectively discriminates between healthy, dystrophic, and transplanted dog samples. Microspectroscopy showed that dystrophic dog muscle displays higher and lower autofluorescence due to collagen cross-linking and NADH respectively than that of healthy and transplanted dogs, defining biomarkers to evaluate the impact of cell transplantation. Our findings demonstrate that DUV radiation is a sensitive, label-free method to assess the histopathological status of dystrophic muscle using small amounts of tissue, with potential applications in regenerative medicine.

## Introduction

Muscular dystrophies (MD) are a genetically heterogeneous group of more than 50 neuromuscular diseases involving skeletal muscle. They all share progressive muscle weakness and wasting but vary in clinical presentation and severity of the symptoms^1,2^. They are characterized by local or generalized muscle fiber degeneration^1^. Duchenne muscular dystrophy (DMD) is the most common MD, affecting 1 per 3500–5500 male newborns^3^. This X-linked recessive fatal muscle disease is caused by mutations in the dystrophin gene, leading to a lack of a functional protein essential to maintain muscle fiber integrity^4^. This results in profound disruption of tissue homeostasis characterized by changes in fiber composition, increased variation in fiber size, centronucleation, inflammation, and replacement of muscle fibers with fibrous and adipose tissues^5,6^.

Histological characterization of the extended changes in dystrophic skeletal muscle provides important information on disease pathophysiology, and is a key tool used to evaluate the effects of novel therapeutic strategies^7^. Standard quantifiable tissue parameters, including the number of fibers, Feret diameter, fiber type composition, percentage of centronucleated fibers, and the proportion of fibrosis and adiposis, are measured in skeletal muscle cross-sections stained using classical topographic staining techniques such as hematoxylin–eosin-saffron (HES), Picrosirius red, and Gomori trichrome, and/or specific immunolabeling^8^. However, evaluation of connective tissue remodeling can sometimes be more challenging: endomysial fibrosis may be difficult to quantify precisely if the surface area occupied is strictly considered without integrating the collagen arrangement within the extracellular matrix, that has been shown to be critical in mechanical function^9^. Therefore, it may not be a sufficiently discriminative criterion for fine assessment of disease progression. Methods to evaluate the efficacy of therapeutic strategies are similarly lacking^10^. Recent evidence suggests that collagen network organization, and in particular collagen cross-linking, constitutes a potential biomarker of DMD^11–13^. At the functional level, collagen cross-linking plays a critical role in muscle stiffness^14,15^, and has been analyzed using polarized light microscopy^12^ and second harmonic generation (SHG) imaging^12^ in tissue sections stained with Herovici^13^ and Picrosirius red. We recently showed that SHG imaging of cleared tissue is a powerful label-free approach for 3D characterization of cardiac fibrosis in DMD^16^. Moreover, autofluorescence of bio-macromolecules in response to Synchrotron deep ultraviolet (DUV) microscopy may constitute a useful marker for ultrastructural characterization of different animal tissues^17–19^ and for monitoring muscle fiber typing and metabolic status^20,21^.

In the present study, we investigated whether dystrophic muscle has a distinct tissue signature that can be detected without chemical staining and without specifically targeting a tissue compartment using spectral signals generated with Synchrotron DUV fluorescence radiation. Skeletal muscle samples from Golden Retriever Muscular Dystrophy (GRMD) dogs, a clinically relevant animal model of DMD^22,23^, were subjected to Synchrotron DUV radiation and the resulting autofluorescence compared with that from healthy Golden Retriever (GR) and GRMD dogs that had undergone adult stem cell (MuStem cells) transplantation (GRMDT), a candidate therapy for DMD^10,24–27^. DUV autofluorescence generated by collagen cross-linking, tyrosine, elastin, tryptophan, and nicotinamide adenine dinucleotide dehydrogenase (NADH) can be identified using selective emission filters of 300–540 nm with narrow band pass (40–60 nm range) and high spectral resolution (step of 0.5 nm) using widefield DUV microscopy and DUV microspectroscopy, respectively^18–21,28^. Using manual image annotation, multivariate statistical analysis without pre-processing of Synchrotron images, and machine learning, we found that the 3 distinct dog groups could be distinguished based on the differential autofluorescence emission intensity in muscle material, corresponding mainly to differences in collagen cross-linking, as measured by widefield DUV microscopy. Importantly, machine learning was here applied from labeled examples to find structural patterns, suitable for automatic classification, processing raw images data entirely instead of segmented elements as edges. Moreover, DUV microspectroscopy revealed two key differences in autofluorescence in naïve GRMD dogs versus both healthy GR controls and MuStem cell-treated GRMD dogs: a significant increase of autofluorescence in the 305–480 range in endomysial connective tissue due to collagen cross-linking, and a decrease of autofluorescence around 460 nm inside muscle fibers due to NADH.

Our findings show for the first time that analysis of Synchrotron DUV radiation enables characterization of the remodeling of dystrophic muscle with a high discriminating power, using only a small quantity of unlabeled tissue. They reveal that this technique, although applied to a small area of muscle which may represent a limit in terms of representativeness and requiring specific microscopy equipment, could be useful for evaluating the efficacy of new therapeutic strategies and assisting patient monitoring in a regenerative medicine context.

## Results

### Intravenous delivery of MuStem cells results in global improvement in clinical status in GRMD dogs

We previously demonstrated that intra-arterial (IA) injections of wild-type MuStem cells in young GRMD dogs results in beneficial effects on clinical status and tissue organization^10,25^. In the present study, we reproduced these effects following intravenous (IV) cell delivery, a surgical approach that is much more applicable in DMD patients, on animals aged 2.8 and 4.4 months (Table 1). Locomotor disability of GRMD dogs was quantified using a composite clinical score based on 17 individual parameters. Healthy GR dogs were assigned a score of 100%. At protocol inclusion, all GRMD dogs had an equivalent clinical score between 89.1 and 93.9%, except for one dog (#6) with a score of 79.8% (Table 2). At the start of the transplantation protocol, GRMD dogs had a close clinical score between 82.0 and 92.1%, except for two dogs (#3 and #7) with a score of 74.7% and 75.1%. At the end of protocol (i.e., 4.9–6.4 months after first cell transplantation) GRMD dogs showed severe clinical impairments, mainly characterized by stiffness, abnormal stance, and inability to run or jump compared to 8–10-month-old healthy GR dogs. The 4 GRMD dogs had variable scores ranging from 23.3 to 72.6%. In return, the scores obtained for GRMDT dogs were much more homogeneous, ranging from 75.1 to 87.0%. A significant difference in locomotor disability score was observed between GRMD and GRMDT dogs (Mann–Whitney test, *p* = 0.029). In addition, the difference between this terminal score and the clinical score obtained at the age of inclusion was calculated to reflect the disease progression in the two dystrophic dog groups. During this time period, all GRMD dogs showed a marked or severe impairment in their clinical scores (median value = -30.9%; range: -51.8 to -19.53%), in contrast to the GRMDT dogs for which clinical scores were much more constant (median value = -4.1%; range: -11.0 to + 1.4%). These results indicate that IV administration of allogeneic MuStem cells has a positive clinical impact, resulting in stabilization of clinical status, consistent with our previous findings following IA transplantation.

**Table 1:** Summary of dog experiments. Each dog (Golden Retriever, GR; Golden Retriever Muscular Dystrophy, GRMD; MuStem cell-treated GRMD, GRMDT) was assigned an identification number. The age of dogs at sacrifice, initiation of immunosuppression and 3 cell injections is expressed in weeks. Cause of death, the nature and status (none or continuous) of immunosuppressant treatment, number of cells injected, and duration of transplantation protocol are indicated. Details of the experiments carried out in each of the 12 dogs are provided. CsA, Cyclosporin A (Neoral®; Novartis); MMF, mycophenolate mofetil (CellCept®; Roche); IF, immunofluorescence; DUV, deep ultraviolet; wDUV, widefield DUV; sDUV, spectroscopy DUV. “X” indicates the experiments that have been carried out. “*” indicates that a score of 100% is obtained by all healthy GR dogs.

Animals				Immunosuppression regimen		Cell transplantation		Clinical follow-up	Tissue analysis			
Group	ID number	Age at sacrifice	Cause of death	Drugs used	Age at initiation	Cell number (10^7^/kg)	Age at different injections	Scoring	Histology staining	Deep ultraviolet radiation Synchrotron		
										IF	wDUV	sDUV
GRMDT	1	40.0	End of protocol	CsA/MMF	16.6	7.4; 8.0; 7.1	17.6; 20.1; 22.0	X	X	X	X	X
	2	40.1	End of protocol	CsA/MMF	18.0	7.8; 5.5; 7.2	19.0; 20.7; 22.4	X	X	X	X	X
	3	39.7	End of protocol	CsA/MMF	11.1	8.0; 6.7; 7.7	12.1; 13.6; 15.1	X	X	X	X	X
	4	37.9	End of protocol	CsA/MMF	15.1	6.7; 5.8; 6.9	16.1; 17.7; 19.1	X	X	X	X	
GRMD	5	47.6	End of protocol	CsA/MMF	18.7	None	–	X	X	X	X	X
	6	37.9	End of protocol	CsA/MMF	10.7	None	–	X	X	X	X	X
	7	33.6	Ethical reason	CsA/MMF	10.6	None	–	X	X	X	X	X
	8	47.4	End of protocol	CsA/MMF	17.6	None	–	X	X	X	X	
GR	9	41.5	End of protocol	None	–	–	–	None; *	X	X	X	X
	10	41.5	End of protocol	None	–	–	–	None; *	X	X	X	X
	11	36.0	End of protocol	None	–	–	–	None; *	X	X	X	X
	12	36.1	End of protocol	None	–	–	–	None; *	X	X	X

**Table 2:** Clinical follow-up of dystrophic dogs. The clinical score of the naïve Golden Retriever Muscular Dystrophy (GRMD) and MuStem cell-treated GRMD (GRMDT) dogs was determined weekly by semi-quantitative evaluation of 17 individual clinical parameters. The evolution of the clinical score was determined between the 1st transplantation and the animal's sacrifice. The age of each dog at sacrifice is expressed in weeks.

Animals			Clinical score			
Group	ID number	Age at sacrifice	At inclusion (%)	At 1st injection (%)	At sacrifice (%)	Evolution during the experiment (%)
GRMDT	1	40.0	90.0	87.3	76.2	−11.0
	2	40.1	89.1	88.3	87.0	−1.2
	3	39.7	89.5	74.7	76.1	1.4
	4	37.9	89.2	82.0	75.1	−6.9
GRMD	5	47.6	93.9	92.1	72.6	−19.5
	6	37.9	79.8	83.7	41.4	−42.3
	7	33.6	90.0	75.1	23.3	−51.8
	8	47.4	91.9	90.8	71.2	−19.6

### MuStem cell delivery in GRMD dogs results in increased muscle regeneration and no reduction in fibrosis

**Figure 1:**
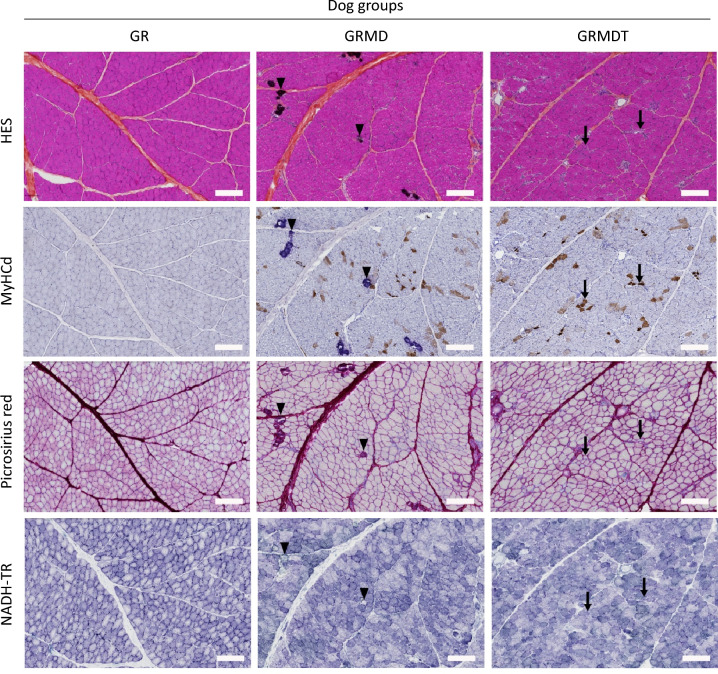
Representative serial histologic sections from dog *Biceps femoris* muscle. Transverse sections from healthy (Golden Retriever; GR), dystrophic (Golden Retriever Muscular Dystrophy; GRMD) and MuStem cell-treated dystrophic GRMD (GRMDT) dogs were stained with hematoxylin–eosin-saffron (HES; first row, arrowheads indicate mineral deposits in GRMD dogs) and immunolabeled for the developmental isoform of myosin heavy chain (MyHCd; second row, arrows indicate groups of positive fibers in GRMDT dog muscle). Connective tissue was visualized by specific Picrosirius red staining (third row). Sections were histoenzymologically stained to visualize nicotinamide adenine dinucleotide dehydrogenase-tetrazolium reductase (NADH-TR; bottom row) activity in mitochondria. The same labels (arrow, arrowhead) have been used on the four histological preparations (upper and lower panels) to highlight the features of interest that are preserved after serial sectioning of the tissues. Scale bars: 100 µm.

To investigate the histopathological effects of IV delivery of MuStem cells in GRMD dog muscle, we performed standard topographical staining and immunolabelling against the developmental isoform of the myosin heavy chain (MyHCd) in cross-sections from GR, GRMD, and GRMDT dogs. Hematoxylin–eosin-saffron (HES) staining showed that the size of individual muscle fibers from GRMD and GRMDT dogs was more heterogeneous across whole muscle sections, compared to healthy GR ones, a change named anisocytosis (Fig. 1; first row). Whole muscle sections from GRMD and GRMDT dogs showed some isolated necrotic fibers, which in GRMD dogs were sometimes replaced by mineral deposits. While no MyHCd^+^ fibers were observed in healthy GR dog muscle sections, some isolated positive fibers were detected in GRMD dog sections (Fig. 1; second row). By contrast, in GRMDT dog muscle sections we observed groups of MyHCd^+^ fibers (arrow in Fig. 1; second row, right panel), indicating more sustained muscle regeneration. In addition, in GRMD and GRMDT dogs, clusters of small cells (about 15–20 µm) with abundant eosinophilic cytoplasm and large nucleus were also detected as positive for MyHCd and corresponded to myoblasts, indicating fiber regeneration foci (Figure S1, upper panel). Whole cross-sections stained with Picrosirius red, which is specific for collagen, showed that perimysial and epimysial tissue was thickened by fibrosis in GRMD versus GR dogs (Fig. 1; third row). No differences in the collagen-positive area relative to total section area were observed between GRMD and GRMDT dogs (Figure S1, lower panel). Finally, histoenzymology to detect nicotinamide adenine dinucleotide dehydrogenase-tetrazolium reductase (NADH-TR) activity in mitochondria showed homogeneous distribution of mitochondria-rich and mitochondria-poor fibers in healthy GR dogs (Fig. 1; bottom row). This distribution was markedly altered in GRMD and GRMDT dogs, with some grouping of fiber types. There was no difference between the two groups of animals in terms of mitochondrial distribution.

**Figure 2:**
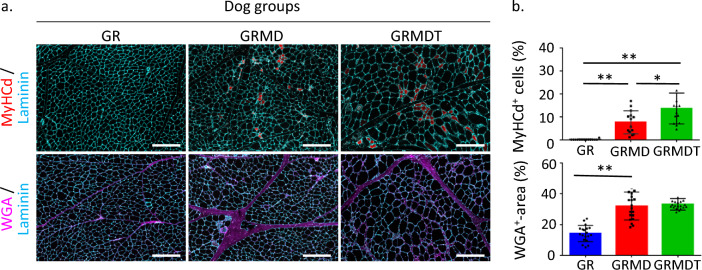
Histomorphometric analysis of dog *Biceps femoris* muscle. (**a**) Regenerative activity was assessed by immunolabeling for the developmental isoform of the myosin heavy chain (MyHCd, top row; red) in muscle cryosections from healthy (Golden Retriever; GR), dystrophic (Golden Retriever Muscular Dystrophy; GRMD) and MuStem cell-treated dystrophic GRMD (GRMDT) dogs. Endomysial connective tissue was stained with wheat germ agglutinin (WGA, bottom row; magenta). Laminin immunolabeling was performed to delimit the outline of muscle fibers. (**b**) The number of MyHCd^+^ fibers and proportion of WGA^+^ stained tissue were determined in all 3 groups. Scale bar: 200 µm. Statistical significance was estimated using a one-way ANOVA followed by a Tukey’s multiple comparison test. ** *p* < 0.0001; * *p* = 0.0046.

To complement these data, we quantified the number of newly formed fibers and connective tissue area in whole muscle sections from the three dog groups based on fluorescent labeling for MyHCd and WGA, respectively (Fig. 2a). We detected 0.04% ± 0.20%, 8.63% ± 5.18%, and 14.40% ± 7.78% MyHCd^+^ fibers in GR, GRMD, and GRMDT dogs, respectively (Fig. 2b, top). GRMDT dog muscle samples showed a higher proportion of MyHCd^+^ fibers compared to GRMD dog samples (*p* < 0.001), indicating greater muscle regeneration following IV transplantation of MuStem cells. Alexa 555-wheat germ agglutinin (WGA) labeling showed that the area occupied by connective tissue corresponded to 14.26% ± 5.20%, 32.05% ± 9.08%, and 33.16% ± 3.74% in skeletal muscle samples from GR, GRMD, and GRMDT dogs, respectively. In GRMD dogs, this area was higher than GR dogs (*p* < 0.001; Fig. 2b, bottom) and similar to that detected in GRMDT dogs, indicating that IV transplantation of MuStem cells does not alter the extent of endomysial fibrosis in dystrophic muscle. Overall, these findings demonstrate that regenerative activity of dystrophic muscle is the primary histopathological marker of the effect of IV MuStem cell administration.

**Figure 3:**
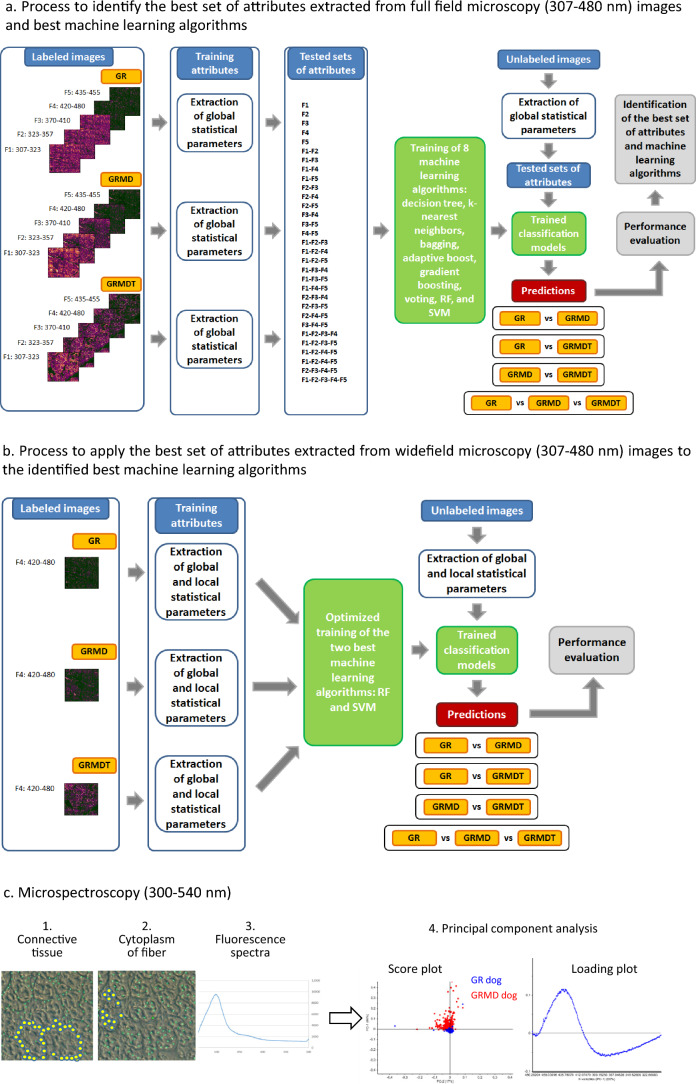
Schematic outline of the experimental protocol and analytical techniques used for label-free imaging of muscle sections using Synchrotron deep ultraviolet radiation. (**a**) Widefield microscopy investigation (307–480 nm). After identifying the different muscle fiber classes (Labeled images), 5 filters were applied to obtain 5 distinct images of each sample, global statistical parameters were calculated using the raw pixel values of those images (Training attributes), and sets of images (Tested sets of attributes) were used to evaluate 8 machine learning models, tested with unlabeled images to select the most appropriate set of attributes and machine learning algorithms. (**b**) Images of filter 4, identified as the best source of attributes were used to extract global and local statistical parameters to test the two best machine learning algorithms, Random Forest (RF) and Support Vector Machine (SVM). (**c**) Microspectroscopy (300–540 nm). A sequential approach was used to measure around fibers (step 1) and inside fibers (step 2), and to produce the corresponding spectra (step 3), after which principal component analysis was performed (step 4).

The marked effects of IV administration of MuStem cells on the general clinical status of GRMD dogs contrasts considerably to the modest effect observed at the histological level. We therefore explored the utility of another imaging modality to determine whether the endogenous fluorescence properties of tissue in response to DUV radiation could serve as a signature of diseased muscle. We applied DUV radiation from the Synchrotron to skeletal muscle samples from each of the 3 dog groups. Specifically, we explored label-free tissue sections using DUV widefield microscopy and microspectroscopy, and performed statistical analysis of fluorescence histograms to generate inputs for machine learning algorithms and principal components analysis methodologies, respectively (as summarized in Fig. 3). Making use of the original raw pixel values, descriptive global statistics were calculated first on images of the 3 dog groups (Fig. 3a) and corresponding filters. Using the resulting dataset, we examined the 4 classification cases that result from combining pairs of dog groups (GR vs GRMD, GR vs GRMDT, GRMD vs GRMDT) and the 3 dog groups together (GR vs GRMD vs GRMDT), to identify initially the best set of attributes and the best machine learning algorithms. Next, the best set of attributes was applied to optimized versions of the identified best machine learning algorithms (Fig. 3b). This experimental approach is well suited to the objectives of developing a non-destructive, low-tissue consumption, label-free method for tissue analysis, using fluorescence analysis without bias.

### Random forest and support vector machine are adequate supervised classification approaches for DUV widefield imaging of muscle samples

**Figure 4:**
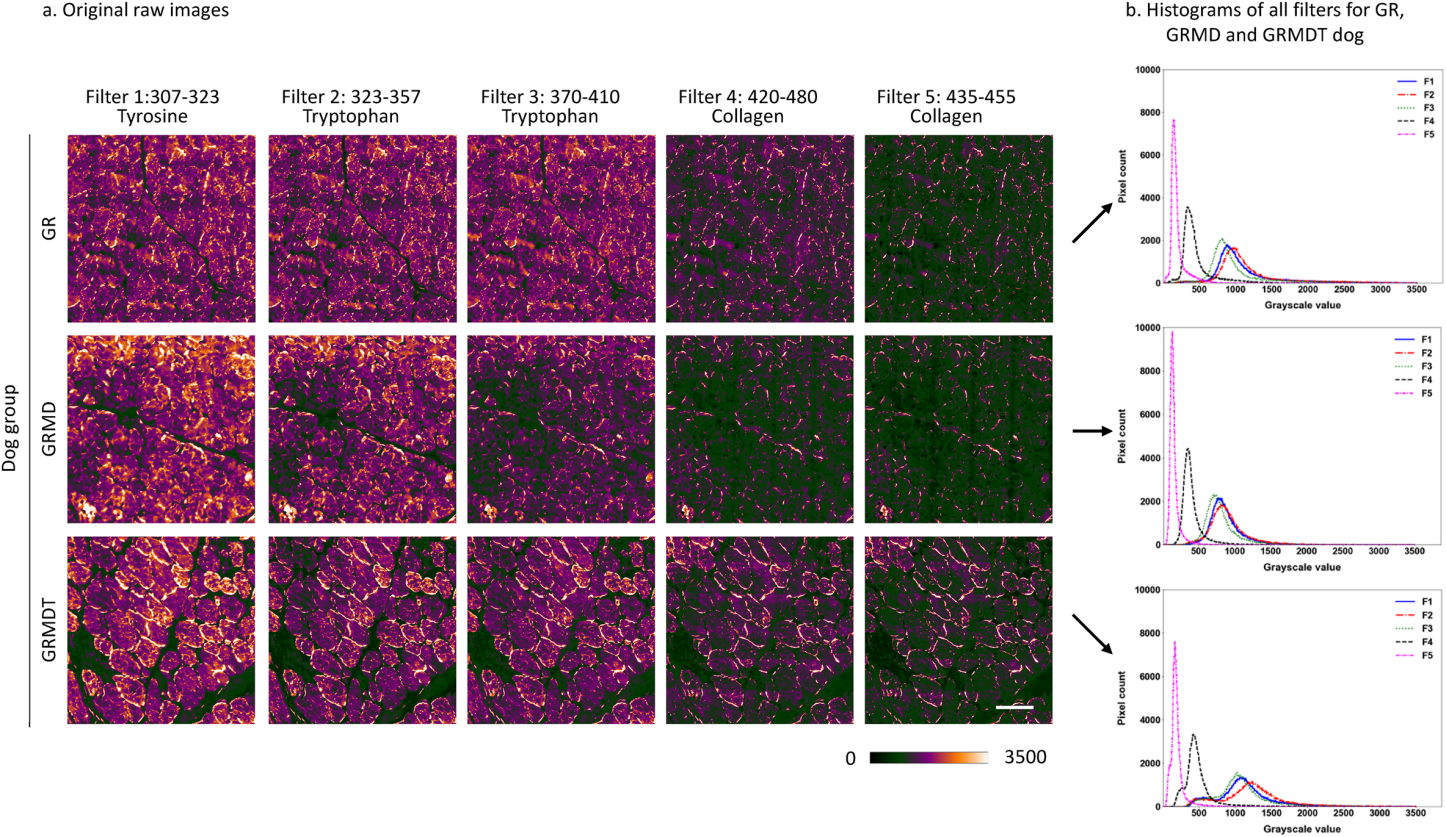
Synchrotron deep ultraviolet radiation for widefield microscopy investigation. (**a**) Examples of original raw images acquired from healthy (Golden Retriever; GR), dystrophic (Golden Retriever Muscular Dystrophy; GRMD) and MuStem cell-treated dystrophic GRMD (GRMDT) dogs using 5 distinct emission filters and different wavelength intervals. (**b**) Corresponding aggregated histograms (represented as an envelope curve of the bins, instead of the conventional set of bins) for all filters and dog groups. Scale bar: 100 µm.

To assess autofluorescence in skeletal muscle tissue, 3 samples from each of the 3 dog groups were exposed to the DISCO imaging beamline (280 nm excitation) for Synchrotron DUV radiation fluorescent microscopy. We selected 5 complementary emission filters to investigate autofluorescence corresponding to tyrosine, tryptophan, and collagen cross-linking. A series of images corresponding to the different patterns of fluorescence mapping obtained with complementary filters was generated for each muscle section (Fig. 4). On the images generated by application of the same contrast, fluorescence intensity is expressed using a color scale ranging from green (low intensity) to orange (high intensity) (blue orange icb lookuptable on Fiji).

**Figure 5:**
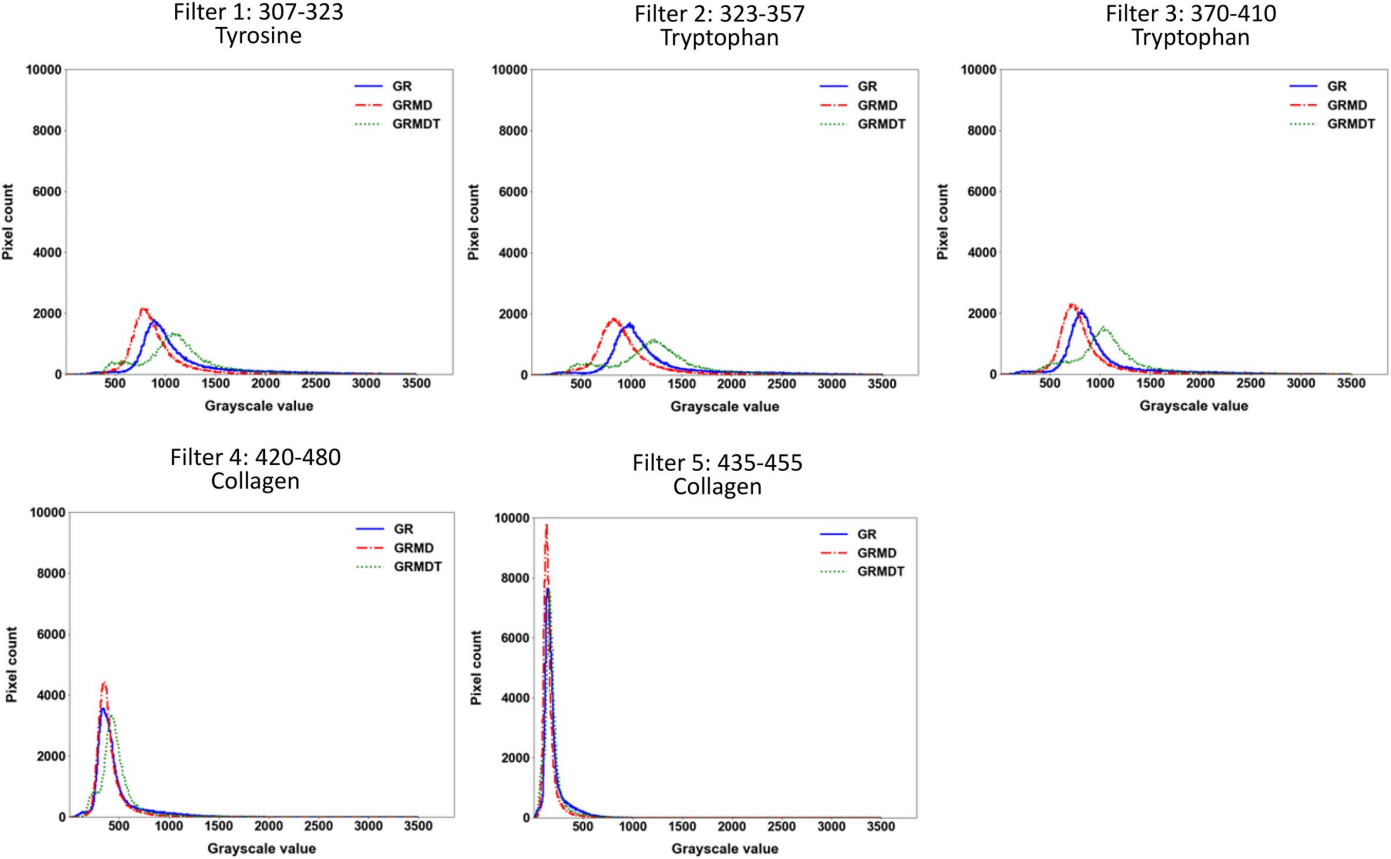
Complementary information representation of Synchrotron deep ultraviolet radiation according to filters. Aggregated histograms of the five filters (represented as an envelope curve of the bins, instead of the conventional set of bins), combining the groups of healthy (Golden Retriever; GR), dystrophic (Golden Retriever Muscular Dystrophy; GRMD) and MuStem cell-treated dystrophic GRMD (GRMDT) dogs for each filter.

**Figure 6:**
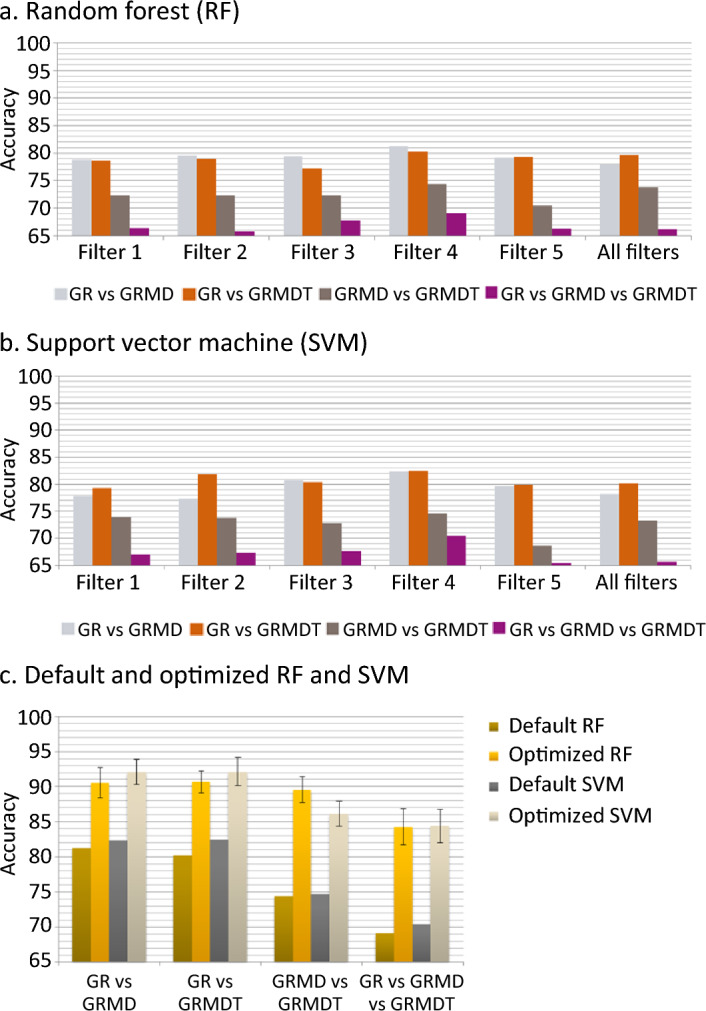
Classification performance (accuracy) for Random Forest and Support Vector Machine. Input for Random Forest (RF; **a**) and Support Vector Machine (SVM; **b**) consisted of global statistical parameters extracted from images generated using each of the 5 filters for each of the 3 dog groups (healthy [Golden Retriever; GR], dystrophic [Golden Retriever Muscular Dystrophy; GRMD] and MuStem cell-treated dystrophic GRMD [GRMDT] dogs) and those extracted from all 5 filters combined. (**c**) Performance classification data obtained for the RF and SVM approaches using as inputs the combination of global and complementary local statistical parameters calculated only for images captured using filter 4 of the 3 dog classes. In both instances (default and optimized parameters), a k-fold cross validation was applied using k = 10. Bars representing standard deviation are shown only for the optimized RF and SVM conditions, as in all the other cases these values were very small (< 0.01%).

As shown in Fig. 4a, the low signal-to-noise ratio and the presence of fuzzy fiber outlines meant that it was not always possible to perfectly isolate the muscle fibers from the connective tissue using conventional image segmentation. Therefore, image analysis was done without applying assumptions or prior knowledge about any features. Statistical characterization of pixel gray level histograms was applied to the images, given their unimodal distributions and the fact that compared quantitative differences can be observed depending on the type of dog and filter. For the 3 dog groups, histograms (depicted as an envelope curve instead of the conventional set of bins) for filters 1, 2, and 3 showed wider variation in gray level intervals and higher gray level values, and their curves were superposed to a large extent and likely to generate redundant information (Fig. 4b). The gray level intervals in the histograms for filters 4 and 5 have lower values and higher counts than filters 1, 2, and 3. Also, when filter histograms were examined separately for the 3 dog groups, filters 4 and 5 produced 2 distinct sets of low fluorescence values, with double the variation observed for filter 4 relative to filter 5 (Fig. 5). These observations suggested that differentiating information could be extracted from the grey level histograms to study the 4 classification cases. Given the histogram morphology elements obtained, we performed image analysis first using a set of 11 global statistical parameters (listed in the “Material and Method” section; Figure S2), testing 8 supervised classification approaches to determine which filter provided the best results. These machine learning approaches corresponded to decision tree, k-nearest neighbors, bagging, adaptive boost, gradient boosting, voting, random forest (RF), and support vector machine (SVM). The statistical parameters were calculated on a total of 13,380 images of 5 filters, with 892 images of each filter type and for each dog group (i.e., when one filter generates attributes, 1784 images are employed to classify 2 classes and 2676 to classify 3 classes). The 4 classification cases were tested applying separately the 8 supervised classification algorithms adapted to small data and to our set of attributes (Fig. 3a). Datasets were balanced and the classification algorithms initially run with default parameters, without optimization. The supervised approaches with the best classification accuracy compared to the other 6 classification algorithms were RF and SVM, respectively (Fig. 6a and b). RF uses a combination of random sampling, feature randomness, and ensemble learning to analyze data. An ensemble of independent decision trees is built on a random subset of training data – to increase diversity and reduce overfitting – and features – to prevent dominant features from overpowering the model. Decision trees are then constructed splitting data recursively by optimizing the homogeneity of the resulting subsets. A classification results from the aggregation of individual trees outputs, i.e., the most common class predicted by the trees is selected. SVM is defined in a high-dimensional feature space of linearly or nonlinearly separable data, where each data point is represented by a vector of feature values. A hyperplane that separates the data of interest into different classes is defined. It is characterized by a margin that represents the maximum distance between the hyperplane and the nearest data points from each class. Support vectors are the data points that lie closest to the decision boundary. The trained model predicts the class of new data points, by evaluating which side of the decision boundary they fall on.

The RF algorithm showed the greatest accuracy for the GR vs GRMD (81.23%) case, followed by GR vs GRMDT (80.27%), GRMD vs GRMDT (74.40%), and GR vs GRMD vs GRMDT (69.10%). The SVM algorithm showed the greatest accuracy for GR vs GRMDT (82.43%), followed by GR vs GRMD (82.35%), GRMD vs GRMDT (74.63%), and GR vs GRMD vs GRMDT (70.42%). The values obtained with SVM were slightly higher than those obtained with RF. Classification of the 25 possible combinations of 2, 3, 4, and 5 filters showed that filter 4 outperformed all other options. Comparison of the 4 classification cases revealed that GR vs GRMD dogs and GR vs GRMDT dogs were likely to be the most contrasted classes regardless of filter type: depending on the classification approach used, the best results always corresponded to one or other of these cases. The lowest accuracy results were obtained for the GR vs GRMD vs GMDT case, independent of the classification algorithm used. Intermediate classification performance was observed for the GRMD vs GRMDT case. Next, the RF and SVM classification algorithms were optimized and run for the same 4 classification cases using the global and complementary local statistical parameters obtained from images generated with filter 4. Each classification algorithm was optimized by automatically scanning intervals of values to identify the specific values of selected parameters that resulted in the best performance. All the possible combinations were evaluated on the resulting assessment grid. For RF, the selected evaluated parameters were number of trees, maximal depth, and criterion. For SVM the parameters evaluated were ν (regularization), ϵ (tolerance to errors), γ (curvature of the decision boundary), and C (penalty of misclassification), using a C-Support Vector Classification (C-SVC) SVM type and a Radial Basis Function (RBF) kernel. Compared to algorithms run using default parameters, optimization resulted in improvements in classification performance from 9.30% ± 2.13% to 15.19% ± 2.52%, translating to a considerable increase in accuracy (Fig. 6c). This confirmed the validity of applying RF or SVM to the combination of global and complementary local statistical parameters for images generated by filter 4. Improvements in performance were observed for all 4 classification cases, as follows: GRMD vs GRMDT (15.19% ± 1.80%) and GR vs GRMD vs GRMDT (15.19% ± 2.52%) for RF, followed by GR vs GRMD vs GRMDT (13.98% ± 2.37%) and GRMD vs GRMDT (11.51% ± 1.79%) for SVM. Improvements in classification performance (between 9.30 and 10.43%) were also observed for GR vs GRMD and GR vs GRMDT (92.11%) with SVM, and GR vs GRMD (90.53%) and GR vs GRMDT (90.70%) with RF. SVM outperformed RF in all 4 cases when default parameters were applied. However, SVM performed slightly better than RF in 2 cases (GR vs GRMD and GR vs GRMDT) when optimization was applied. When optimized, both algorithms performed comparably in the GR vs GRMD vs GRMDT case. These findings establish that widefield DUV microscopy analysis with low spectral resolution (60 nm) can efficiently discriminate GR, GRMD, and GRMDT dog muscle samples based on autofluorescence emitted at 420 nm and 480 nm.

### DUV fluorescence microspectroscopy with high spectral resolution effectively distinguishes muscle samples from the 3 dog groups

**Figure 7:**
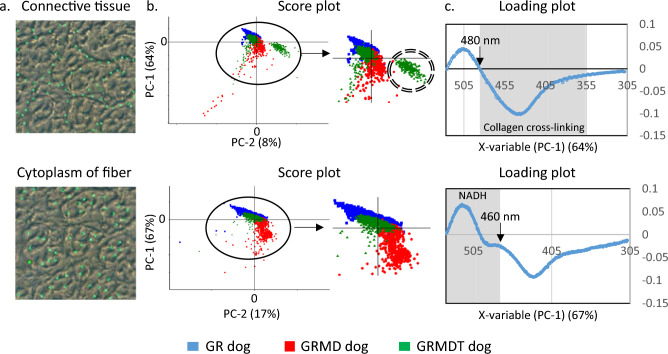
Investigation of connective tissue and muscle fiber cytoplasm with Synchrotron deep ultraviolet radiation microspectroscopy. (**a**) Deep ultraviolet (DUV) spectral data were obtained between 300 and 540 nm for connective tissue (top) and fiber cytoplasm (bottom) in skeletal muscle of 3 animals per dog group: healthy [Golden Retriever; GR], dystrophic [Golden Retriever Muscular Dystrophy; GRMD] and MuStem cell-treated dystrophic GRMD [GRMDT]. (**b**) Data were analyzed by Principal Component Analysis (PCA), pre-processed (unit vector normalization) and subjected to multivariate data analysis (The Unscrambler® X; CAMO Software Process AS). The results, obtained for healthy GR (blue), GRMD (red) and GRMDT (green) dogs, were represented by score plot in which each dot corresponds to a single spectrum. In connective tissue (top), 3 clusters were separated in the score plots: (i) along the PC-1 axis, separation of GRMD dogs (red; located on negative part of PC-1) from healthy GR dogs (blue) and part of GRMDT dogs (green), both being located on positive side of PC-1 axis; (ii) along PC-2 axis, GRMD dogs (red) and part of GRMDT dogs (green; surrounded) which formed a cluster at right of PC-2 axis. Healthy GR dogs (blue) and part of GRMDT dogs (green) were distributed from either side of PC-2 axis. In cytoplasm of muscle fiber (bottom), 2 clusters were separated: (i) along the PC-1 axis, separation of GRMD dogs (red; located on negative part of PC-1) from healthy GR dogs (blue) and one GRMDT dog (green), both merged and located on positive side of PC-1 axis; (ii) along PC-2 axis, GRMD dogs (red) formed a cluster at right of PC-2 axis whereas most of healthy GR dogs (blue) and GRMDT dogs (green) were distributed from either side of PC-2 axis. (**c**) Loading plots, where the contribution of each wavenumber in the clustering of the spectra was investigated, were also generated. The corresponding loading plots revealed the main characteristic emission bands of collagen cross-linking (top) and NADH (bottom).

Based on the encouraging results obtained with widefield DUV microscopy, we sought to refine the spectral signature of muscle from the 3 dog groups by using Synchrotron DUV microspectroscopy. Although much more time consuming, this approach allows for a level of spectral resolution 40 times higher than that obtained in widefield microscopy separate scanning of muscle fibers and connective tissue, thanks to the scan by point function done on image obtained in brightfield microscopy. We used this technique to analyze the connective tissue and cytoplasm of muscle fibers with a spectral resolution of 0.5 nm (Fig. 7a). DUV spectral data were acquired in the 300–540 nm range in muscle samples from 3 healthy GR (blue), 3 GRMD (red), and 3 GRMDT (green) dogs. Spectra were pre-processed and underwent multivariate data analysis (The Unscrambler® X, CAMO Software Process AS). Principal Component Analysis (PCA) was performed and the results were represented in a score plot in which each point corresponds to a single recorded spectrum (Fig. 7b). In connective tissue (top), 3 clusters can be distinguished along the PC-1 axis (64%) in the score plot. While blue spots (GR) formed a homogeneous cluster above the horizontal axis, red spots (GRMD) formed a large heterogeneous grouping along the vertical negative axis. Interestingly, a cluster of green spots (GRMDT) merged with the blue cluster (GR), while a second cluster of green spots located below the horizontal axis was clearly separated from the red spots (GRMD). The score plot obtained for cytoplasm of muscle fibers revealed more pronounced separation of the dystrophic dog groups. Three clearly distinct clusters corresponding to the different dog groups were observed along the PC-1 axis (67%). Green spots (GRMDT) were largely separated from red spots (GRMD) and showed a high level of overlap with blue spots (GR). We next examined the loading plot, in which the contribution of each wavenumber to the clustering of the spectra was investigated from the positive or negative position relative to the x-axis of the loading plot (Fig. 7c). For measurements in connective tissue, we showed that collagen cross-linking band such as pentosidine (355–480 nm) was in the negative portion of the loading plot (below x axis), indicating greater fluorescence intensity for GRMD (red spots) than GR (blue spots) dogs in an emission range known to be a signature of collagen cross-linking^19^. Interestingly, this was also the case with the comparison with GRMDT dogs (green spots), except for one animal (ID#2; cluster surrounded). Moreover, the characteristic NADH band (540–480 nm) was in the positive portion of the loading curve (above the x-axis), indicating lower NADH fluorescence intensity for GRMD (red spots) versus GR dogs. Except for one animal, GRMDT dogs showed higher NADH fluorescence intensity than GRMD dogs. For measurements taken within fibers, the characteristic emission band of NADH was located in the positive portion of the curve (above x axis) indicating comparable fluorescence intensity in GRMDT (green spots) and GR (blue spots) dogs, both of which were higher than that of GRMD dogs (red spots). The spectral feature observed at 460 nm in the loading plot further supports the view that NADH autofluorescence can effectively discriminate GRMD dog muscle samples from those of GR and GRMDT dogs. Overall, these findings indicate that DUV microspectroscopy data can be used to distinguish GRMD dogs from the 2 other groups based on lower NADH fluorescence intensity (540–480, 460 nm) within muscle fibers and higher collagen cross-linking-associated fluorescence intensity (480–355 nm) in connective tissue. Importantly, we show for the first time a clear separation between GRMDT and GRMD dog clusters, with a first cluster encompassing connective tissue data points for 2 of 3 GRMDT dogs and 3 GR dogs and a second cluster encompassing cytoplasm fiber data points for all GRMDT and GR dogs. These findings further underscore the beneficial impact of MuStem cell transplantation on the muscle phenotype of GRMD dogs.

## Discussion

In this study, we provide original and compelling data demonstrating the power of DUV radiation to discriminate damaged skeletal muscle tissue, highlighting its potential for characterizing dystrophic tissue remodeling. Our data indicate that a statistical analysis of endogenous fluorescence intensity histograms collected at different wavelengths from DUV widefield measurements is a valid unbiased approach to process the totality of the signal generated by the tissue section. Indeed, this approach, performed for the first time on a Synchrotron dataset, allowed us to distinguish skeletal muscle of naïve GRMD dogs from that of GRMD dogs that had undergone adult stem cell transplantation, in contrast to the results obtained using conventional quantitative histological techniques. Furthermore, the discriminatory power of DUV widefield data was confirmed using DUV microspectroscopy, which is characterized by a high level of spectral resolution, validating the experimental approach. We used DUV radiation images to distinguish with high level of accuracy for the first time the following groups: GR vs GRMDT, GRMD vs GRMDT, and GR vs GRMD vs GRMDT, apart from the already well-classified GR vs GRMD dog groups. Despite difficulties correctly determining the shape of fibers by image segmentation and therefore the impossibility of classifying fibers according to geometrical characteristics, these results suggest that combined global and local statistical parameters provide suitable attributes for supervised machine learning. At a biological level, we showed that MuStem cell transplantation in an animal model of dystrophy can lead to an organization of the collagen network more similar to that seen in healthy animal. Together, these findings describe a new modality of microscopic examination of dystrophic muscle samples which requires very little tissue and is label-free and highly sensitive, allowing for more in-depth analysis of tissue remodeling. In addition, the results provide further evidence of the ability of MuStem cells to favorably remodel dystrophic muscle over the long-term following systemic delivery, with an impact observed here in particular on the connective tissue 5 months after administration. This reinforces the idea of a pleiotropic action of these adult stem cells, as evoked in our previous studies involving unbiased Omic approaches^25,26^. Overall, our findings further consolidate MuStem cell transplantation as a promising candidate therapy for DMD.

A key attribute of DUV radiation microscopy/microspectroscopy is the absence of labeling with external probes (i.e., dyes and antibodies), which can be sources of experimental and/or species-specific artifacts. This gives the technique a universal character that better lends itself to the comparison of studies performed in different laboratories. A second key benefit of the technique is that it requires a small amount of tissue material, which is commonly a limiting element, particularly when working with human samples. The downside of exploring a restricted area of muscle is that it may not be representative of the state of the tissue whether in preclinical studies or clinical trials, and the idea of exploiting several areas in parallel needs to be taken on board to ensure that the data produced is a true reflection of the tissue's overall condition.

Using DUV widefield microscopy and microspectroscopy, we showed that analysis of endogenous fluorescence emitted by connective tissue can distinguish GR, GRMD, and GRMDT dog muscle with a high level of accuracy. This is particularly interesting given that endomysial fibrosis is one of the main histopathological features in the evolution of DMD^29–31^. The area occupied by connective tissue in cross-sections of dystrophic muscle, previously stained with Picrosirius red, WGA or immunolabelled for collagen I^32–34^, is routinely quantified to acquire information on disease progression and/or assess the impact of gene or cell therapy strategies in preclinical studies^10,35,36^. Our findings, generated from DUV radiation-based approach carried out for the first time on dystrophic dog muscle, consolidate the previous results revealing that the organization of the collagen network is, as well as the fibrosis area, an important criterion to consider when studying the remodeling of dystrophic muscle. Interestingly, analysis of DUV radiation targeting the connective tissue framework allowed distinction of GRMDT vs GRMD muscle, while no significant differences were found when the endomysial tissue area was considered through conventional histopathological approaches. This suggests a higher sensitivity of the DUV approach. These original data were obtained here on the *Biceps femoris* muscle, so it would be informative to define whether identical results are found in other dystrophic muscles that may present a different lesion phenotype, in order to inform the degree of discrimination of the measurement. It is also worth noting that qualitative changes in fibrous tissue (i.e., degree of collagen cross-linking) have been reported to play a major functional role in dystrophic muscle^14,15^, further highlighting the relevance of the DUV radiation data presented here. For example, the architectural organization of the collagen network is a key contributor to muscle stiffness in DMD patients as well as the *mdx* mouse and GRMD dog models^11,12^. A recent study using polarized light microscopy or second harmonic generation microscopy reported no clear correlation between the level of collagen fiber alignment and the muscle elasticity criterion^12^. Based on the differential spectral data obtained for GRMD and GRMDT dogs, it would be interesting to perform functional exploration and a DUV radiation analysis using the same isolated muscles to investigate the association between connective tissue modifications caused by MuStem cell transplantation and muscle stiffness and elasticity. Using DUV microspectroscopy to perform high resolution spectral measurements in connective tissue, we obtained spectra for 2 of the 3 GRMDT dogs similar to those obtained in GR dog samples, which may suggest a variable response to cell transplantation. Nonetheless, it should be noted that the measurements were performed on a single muscle type, the *Biceps femoris* muscle, and there was no correlation with the results obtained for the other criteria evaluated i.e.*,* clinical score and regeneration activity. In agreement with several studies reporting phenotypic variability in GRMD dogs^25,37^, this result could also reflect more pronounced lesion intensity in 1 of the GRMD dogs included in the study.

Apart from collagen cross-linking, tryptophan, tyrosine and NADH are among the most abundant autofluorescent molecules observed in muscle exposed to DUV radiation^19,38^. Another key finding of our study is that spectral data corresponding to NADH in the cytoplasm of muscle fibers clearly distinguish GRMDT from GRMD dog muscle, but also group them with those of GR dog suggesting a return to normal value for this parameter following cell delivery protocol. These results indicate that DUV autofluorescence intensity associated with NADH within muscle fibers could constitute a second biomarker of the beneficial impact of MuStem cell transplantation.

DUV microspectroscopy measurements performed at the level of connective tissue and muscle fiber cytoplasm indicate a significant increase in autofluorescence associated to collagen cross-linking and a concomitant significant decrease in NADH autofluorescence intensity in muscle from GRMD versus GR dogs. These observations are consistent with the findings of several studies showing that modification of the collagen cross-linking^11,39^ and mitochondrial dysfunction^40,41^ are associated with DMD. We recently demonstrated significant disorganization of the collagen network in cardiac muscle from dystrophic rats, as evidenced by fiber poly-orientation, compaction, and shortening^16^. In the present study, we report changes in collagen cross-linking and in the NADH, which is predominantly from the mitochondria where it serves to ATP synthesis, in GRMD muscle following systemic delivery of MuStem cells, with corresponding spectral characteristics similar to those obtained in GR dog muscle. These results are in agreement with our previous Omics studies on the consequences of MuStem cell transplantation, revealing an impact on multiple biological processes, including the structural integrity of muscle bundles via action at the level of fiber architecture, extracellular matrix organization, and energy metabolism^25,26^. More than 6 months after intra-arterial delivery of MuStem cells in GRMD dogs, quantitative proteomic of *Biceps femoris* muscle necropsies has revealed significant changes in the collagen I content and in the enrichment of gene ontology terms, including oxidative-reduction process, ATP metabolic process, and mitochondrion, compared to data obtained in the naïve GRMD dog^26^. Interestingly, different NADH dehydrogenase subunits have been identified as significantly underrepresented proteins after cell transplantation.

Our study demonstrates that DUV radiation microscopy/microspectroscopy represents a powerful tool for label-free exploration of muscle sections for preclinical studies. Nonetheless, certain improvements to optimize Synchrotron DUV acquisition could help further increase its discriminatory power. Image acquisition using dual-optimal excitation wavelengths could allow for more specific investigation of collagen cross-linking and NADH. Moreover, a more specific emission filter band pass would allow greater separation of fluorescence emission and therefore better discrimination between muscles from GR, GRMD, and GRMDT dogs. Given the size of dataset used in the present study, we applied supervised machine learning with feature engineering. However, our results indicate that it is possible to improve classification performance. An alternative to optimized classification models would be to define, automatically and in an unsupervised manner, the appropriate DUV radiation image features by means of representation learning using a deep learning approach like autoencoders. This would require studies using an animal model of DMD that allows for a greater sample set size than can be attained with GRMD dogs, such as the DMD^*mdx*^ rat, which is increasingly used in preclinical studies^35,42^, to increase the size of the image-related dataset.

## Conclusion

We describe the use of DUV radiation combined with widefield microscopy and microspectroscopy to perform the first unbiased analysis of endogenous fluorescence in dystrophic muscle. Our findings show that DUV Synchrotron radiation used in widefield microscopy and microspectroscopy represents a sensitive tool that complements classical histomorphometry approaches and facilitates the characterization of dystrophic muscle and the efficient evaluation of cell-based therapeutic strategies.

## Methods

### Animals

A total of 12 male 2.5-month-old Golden Retriever (GR) dogs were included in this study. All dogs were obtained from the Centre d’Elevage du Domaine des Souches (CEDS, Mezilles, France) or the Boisbonne Center for gene and cell therapy (Oniris, Nantes, France). The dogs were housed at the Boisbonne Center in a controlled environment (temperature 21 ± 1 °C, 12-h light/dark cycle). Golden Retriever Muscular Dystrophy (GRMD) dogs were identified at birth using polymerase chain reaction (PCR)-based genotyping, as previously described^43^. Three groups (N = 4 animals per group) were created for clinical and/or pathophysiological studies (Table 1): GRMD dogs treated with MuStem cells and immunosuppressants (IS) (GRMDT; #1 to #4); GRMD dogs receiving IS only (GRMD; #5 to #8); and healthy dogs (GR; #9 to #12). The study was carried out in accordance with the recommendations of the Guide for the Care and Use of Laboratory Animals of the French National Research Council. The protocol was approved by the Ethics Committee on Animal Experimentation of the Pays de la Loire Region, France (Permit Number: CEEA.2012.104). Furthermore, the study was carried out in line with the ARRIVE guidelines (https://arriveguidelines.org). All surgeries were performed under anesthesia induced with ketamine (Imalgene 1000, Merial, Toulouse, France)/diazepam (Valium, Roche, Boulogne-Billancourt, France) and that was maintained using an inhalational mixture of isoflurane (Vetflurane, Virbac, Magny-en-Vexin, France) and oxygen. To minimize suffering, analgesia treatment was performed with tolfenamic acid (4 mg/kg, Tolfedine, Vetoquinol SA, Magny Vernois, France). Pain was evaluated daily as part of a complete clinical evaluation performed by a veterinarian and analgesia was provided if deemed necessary. Dogs were euthanized by intravenous administration of sodium pentobarbital (2000 mg; Dolethal, Vetoquinol SA, Magny Vernois).

### Isolation of canine MuStem cells

Wild-type MuStem cells, corresponding to delayed adherent stem cells, were isolated from hind limb muscles of 10-week-old healthy dogs, as previously described^10^. Cells were incubated at 37 °C with 5% CO_2_, and passaged every 4 to 5 days when they reached approximately 75% confluence. Growth medium was replaced every 2 days.

### Immunosuppressive treatment

Immunosuppression of GRMD and GRMDT dogs was achieved by daily administration of 27 mg/kg of oral CsA (Neoral®; Novartis, Rueil-Malmaison, France) in combination with 6 mg/kg mycophenolate mofetil (CellCept®; Roche, Paris, France). Ketoconazole (10 mg/kg; Nizoral®; Janseen-Cilag, Issy-les- Moulineaux, France) was also added daily to decrease CsA catabolism. Blood levels of CsA were monitored twice per week and maintained between 250 and 350 ng/mL by individual dose adjustments. The immunosuppressive regimen was started 1 week before MuStem cell administration and maintained throughout the course of the study.

### Systemic delivery procedure

MuStem cells were used at passage 5 (P5) or 6 (P6), which corresponds to cells having undergone between 22 and 27 cumulative population doublings. Cell suspensions were prepared with a density range of 12–18 × 10^6^ cells/mL in 0.9% NaCl / 2.5% homologous serum / 10 IU/mL heparin. Three injections of 5.5–8.0 × 10^7^ cells/kg into the cephalic veins were performed at 10- to 12-day intervals in immunosuppressed GRMD dogs (GRMDT; #1 to #4) beginning at age 11.0–18.2 weeks (Table 1), using laminar flow at a rate of 12–15 mL/min, as previously described^10^.

### Clinical follow-up

Dogs were clinically assessed by a veterinarian in a non-blinded manner on a weekly basis throughout the experimental protocol, using a modified version of the grid previously described^10,43^. Briefly, a clinical score was established based on 11 locomotion and muscle criteria and 6 items related to the general health status. The score was expressed as the percentage of the maximum score of 100% for all healthy dogs.

### Muscle sampling

Small pieces (0.5 cm^3^) of *Biceps femoris* muscle necropsies were collected surgically from the middle portion of the muscle in 36.0- to 47.4-week-old dogs, except for one dog that was euthanized at 33.6 weeks for ethical reasons, and used for histological analyses. This time point corresponds to 5.0–6.7 months after initiation of the cell transplantation protocol. Muscle samples were snap-frozen in isopentane (VWR international, Fontenay-sous-Bois, France), cooled in liquid nitrogen and stored at -80 °C until processing. Serial transverse 8 μm-thick cryostat sections were placed on glass slides for histological staining and fluorescence immunolabeling and subsequent analysis with slide scanner (Axioscan Zeiss, Jena, Germany). In addition, serial transverse 10 μm-thick cryostat sections were placed on quartz slides for deep ultraviolet (DUV) microscopy and DUV microspectroscopy (Synchrotron DISCO beam line, Soleil, France).

### Histology and immunohistochemistry

Serial muscle Sects. (8 μm) were stained with hematoxylin–eosin-saffron (HES), Picrosirius red, and nicotinamide adenine dinucleotide dehydrogenase-tetrazolium reductase (NADH-TR) to provide information on muscle bundle organization, connective tissue content, and mitochondrial distribution. To specifically study endomysial fibrosis, sections were incubated overnight at 4 °C with Alexa-Fluor 555-conjugated wheat germ agglutinin (WGA; 1:500, W32464; ThermoFisher Scientific, Waltham, MA, USA) after permeabilization with 0.1% Triton X-100 (Sigma-Aldrich, Saint Quentin-Fallavier, France) and incubation with blocking buffer (mixture of 5% goat serum and 5% bovine serum albumin in 0.1 M phosphate-buffered saline [PBS], Sigma-Aldrich). To assess muscle regenerative activity, sections were permeabilized and saturated as described above, then incubated overnight at 4 °C with antibody directed against the developmental isoform of myosin heavy chain (MyHCd; 1:100, NCL-MHCd; Novocastra) and Alexa-Fluor 555-conjugated goat anti mouse IgG (1:300, ThermoFisher Scientific, Waltham, MA, USA). For quantification of MyHCd^+^ fibers, 5 regions of interest each comprising 100 to 300 fibers were considered on a whole histological section of each of 4 dogs per group, so as to analyze a set of 500 to 1500 fibers per dog. Histological sections were read and analyzed by a veterinary pathologist certified by the European board committee of the specialty.

### Slide scanner imaging

Images, corresponding to the whole section of each tissue sample, were acquired using a slide scanner (AxioScan.Z1, Zeiss, Jena, Germany) with fluorescence and brightfield imaging modes (Plan Apochromat 10X objective). Brightfield imaging was performed with Led illumination and Tri-CDD Hitachi camera detection. Fluorescence imaging was performed with XCITE LED FIRE illumination, emission Band Pass (EM BP 445/50 (DAPI), EM BP (525/50 [Alexa-Fluor 488], and EM BP 605/70 [Alexa-Fluor 555]). Histomorphometry was performed using Fiji software. The surface area corresponding to WGA fluorescent labelling was measured in the endomysium. The proportion of MyHCd^+^ fibers was quantified in muscle sections from the 4 dogs in each group, considering at least 500 fibers per group. Statistical analyses were performed using GraphPad Prism v6.0 (GraphPad Software, La Jolla, CA, USA).

### Synchrotron DUV radiation

Synchrotron UV fluorescence imaging was performed at the DISCO beamline at the SOLEIL Synchrotron radiation facility (Saint-Aubin, France)^44^. The DISCO Synchrotron beamline has an experimental imaging station on which the bending magnet delivers DUV radiation in the visible, continuously tunable from 180–600 nm (1.2–10 eV). Many aromatic groups and enzymes naturally luminesce under UV excitation without external markers. This makes the DISCO beamline well suited to the in situ study of biomedical samples, through analysis of the intrinsic fluorescence of molecules^45,46^.

### Synchrotron widefield DUV microscopy

#### Image acquisition

Frozen *Biceps femoris* muscle cross-Sects. (10 µm) from 4 GR, 4 GRMD, and 4 GRMDT dogs were analyzed. Sections were placed on quartz slides for DUV imaging. The Synchrotron widefield DUV imaging system is constructed around a Zeiss Axio Observer Z1 (Carl Zeiss) inverted microscope with quartz-only optics. The white beam of the DISCO beamline at Synchrotron SOLEIL is monochromatized by an iHR320 (Jobin–Yvon Horiba) before coupling with the entrance of the modified Zeiss Axio Observer Z1. The monochromatic beam was settled at 280 nm. A sharp dichroic mirror transmitting only above 300 nm (Omega Optical) reflected the incident light before focalization onto the sample through a Zeiss Ultrafluar 40X (N.A. 0.6, glycerin immersion). Emission was recorded with a Pixis 1024-BUV (Princeton Instruments) camera after passing through a series of bandpass filters (307–323 nm, 327–353 nm, 370–410 nm, 420–480 nm and 435–455 nm; Semrock, IDEX Health & Science, LLC). These channels were chosen according to the aromatic groups that fluoresce at these wavelengths, based on which the presence of different amino acids, proteins, or collagen cross-linking can be associated. Thus, the first channel was assimilated to the presence of tyrosine, the second and third to the presence of tryptophan and finally the last two to collagen cross-linking^19^. Fluorescence images were typically recorded with exposure times of 1–5 s. Images were acquired with a 40X objective (0.276 µm/pixels). For stitching purpose, a centered region of interest (404 × 424 pixels corresponding to 111 µm × 117 µm) of the camera has been selected. Therefore, no stitching artefact or shading were observed, allowing the generation of large images comprising 36 smaller images (350 × 350 pixels) (example: 2424 × 2544 pixels = 666 µm × 702 µm). One hundred forty-four images were acquired per sample. Illustrations of DUV widefield microscopy acquisitions were performed by using Fiji software^47^.

#### Image classification

Machine learning algorithms were applied because, to identify the 3 classes of interest, the complex and variable relevant features of autofluorescence images are very difficult to define manually or by applying segmentation algorithms, whereas they can be captured more efficiently by learning approaches. Considering that statistical parameters values of each image only represent one dog class (regardless of the filter type)**,** patterns in the DUV images were evaluated by training supervised classification models adapted to small data scenarios, using labeled examples of each of the 3 classes to analyze 4 classification problems: GR vs GRMD, GR vs GRMDT, GRMD vs GRMDT, and GR vs GRMD vs GRMDT. Global statistical parameters calculated for the histograms of all filters for each of the 3 classes were used to address the 4 classification problems, and 8 supervised classification approaches were tested: decision tree, k-nearest neighbors, bagging, adaptive boost, gradient boosting, voting, support vector machine (SVM), and random forest (RF) (Fig. 3a). Based on the results we identified the filter with the best classification performance, as well as the corresponding classification approaches. Complementary local statistical parameters were then calculated from the histograms of the filter with the best classification performance and combined with the respective global statistical parameters (Figure S2), to be used as inputs in the best performing classification approaches. Machine learning algorithms were developed with the Python programming language and some libraries like OpenCV (open computer vision), NumPy (numerical programming), Pandas (data analysis), scikit-learn (machine learning), PIL (image manipulation), and matplotlib (visualization).

#### Synchrotron DUV microspectroscopy

The tissue sections were placed without specific mounting medium on circular quartz slides for DUV imaging (diameter 12.7 mm, thickness 0.17 mm, ESCO optics, New Jersey, USA). A monochromatic beam was used at 280 nm through a 40X objective (Ultrafluar, Zeiss, Germany) and the recovery of the fluorescence was carried out between 300 and 540 nm with 0.5 nm spectral resolution. The spectrum was chosen until 530 nm to explore the characteristic NADH band^19^. Single point exploration was performed to separately analyze fiber and connective tissue. A total of 230 spectra were acquired per region (fiber cytoplasm and connective tissue) and per animal, using 3 dogs per group (GR, GRMD, GRMDT). Spectral data were analyzed using The Unscrambler® X (CAMO Software Process AS) multivariate dataset. The spectra were grouped in a same matrix. Working matrices were created by gathering samples from the same dog groups. Six hundred ninety spectra per dog group were pre-processed according to the unit vector normalization method in order to be able to compare each spectrum one by one and perform Principal Component Analysis (PCA) on the variance between spectra. While the score plots allowed comparison of the DUV spectra, the corresponding loading plots revealed the main characteristic emission bands behind the clustering of the spectra.

### Statistical analysis

Data are expressed as the mean ± standard deviation (SD). Data were analyzed using a one-way ANOVA followed by Tukey’s multiple comparison test. Statistical analyses were performed using GraphPad Prism v6.0 (GraphPad Software, La Jolla, CA, USA). A *p*-value ≤ 0.05 was considered significant. Global and local statistical parameters for image classification were calculated with the Python programming language and the scipy.stats (statistical functions) library.

## Supplementary Information

Supplementary Information.

## Data Availability

The datasets used and/or analyzed during the current study are available from the corresponding author on reasonable request.
