# Supplementary material for: Specific and label-free endogenous signature of dystrophic muscle by Synchrotron deep ultraviolet radiation
Source: Sci Rep. 2023 Jul 4;13:10808. doi: 10.1038/s41598-023-37762-1 (PMC10319894; doi:10.1038/s41598-023-37762-1)
Supplement: Supplementary file 1 — Supplementary Information. [file 41598_2023_37762_MOESM1_ESM.pptx]

## Slide 1
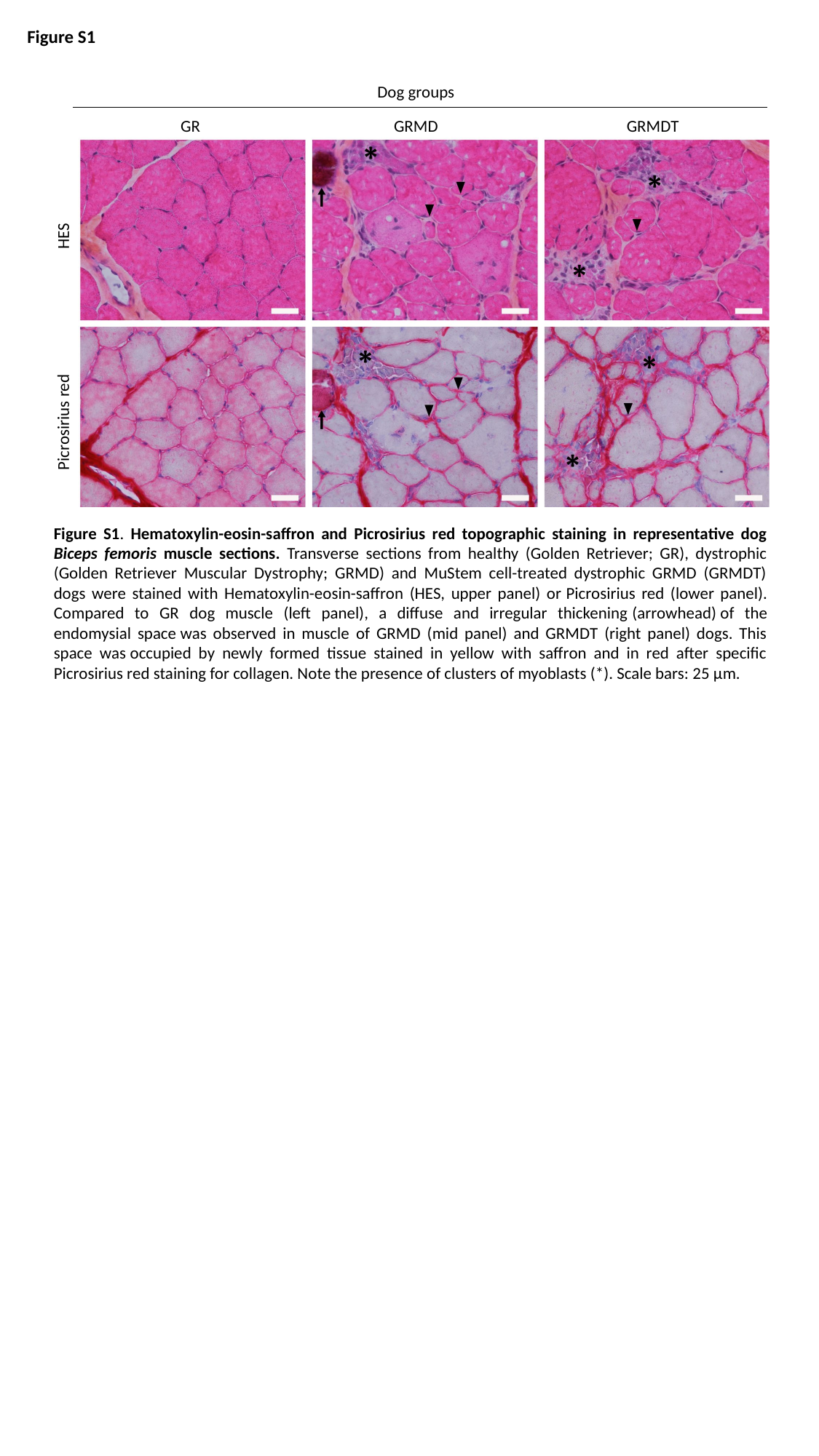

Figure S1
Dog groups
GR
GRMDT
GRMD
HES
Picrosirius red
Figure S1. Hematoxylin-eosin-saffron and Picrosirius red topographic staining in representative dog Biceps femoris muscle sections. Transverse sections from healthy (Golden Retriever; GR), dystrophic (Golden Retriever Muscular Dystrophy; GRMD) and MuStem cell-treated dystrophic GRMD (GRMDT) dogs were stained with Hematoxylin-eosin-saffron (HES, upper panel) or Picrosirius red (lower panel). Compared to GR dog muscle (left panel), a diffuse and irregular thickening (arrowhead) of the endomysial space was observed in muscle of GRMD (mid panel) and GRMDT (right panel) dogs. This space was occupied by newly formed tissue stained in yellow with saffron and in red after specific Picrosirius red staining for collagen. Note the presence of clusters of myoblasts (*). Scale bars: 25 µm.

## Slide 2
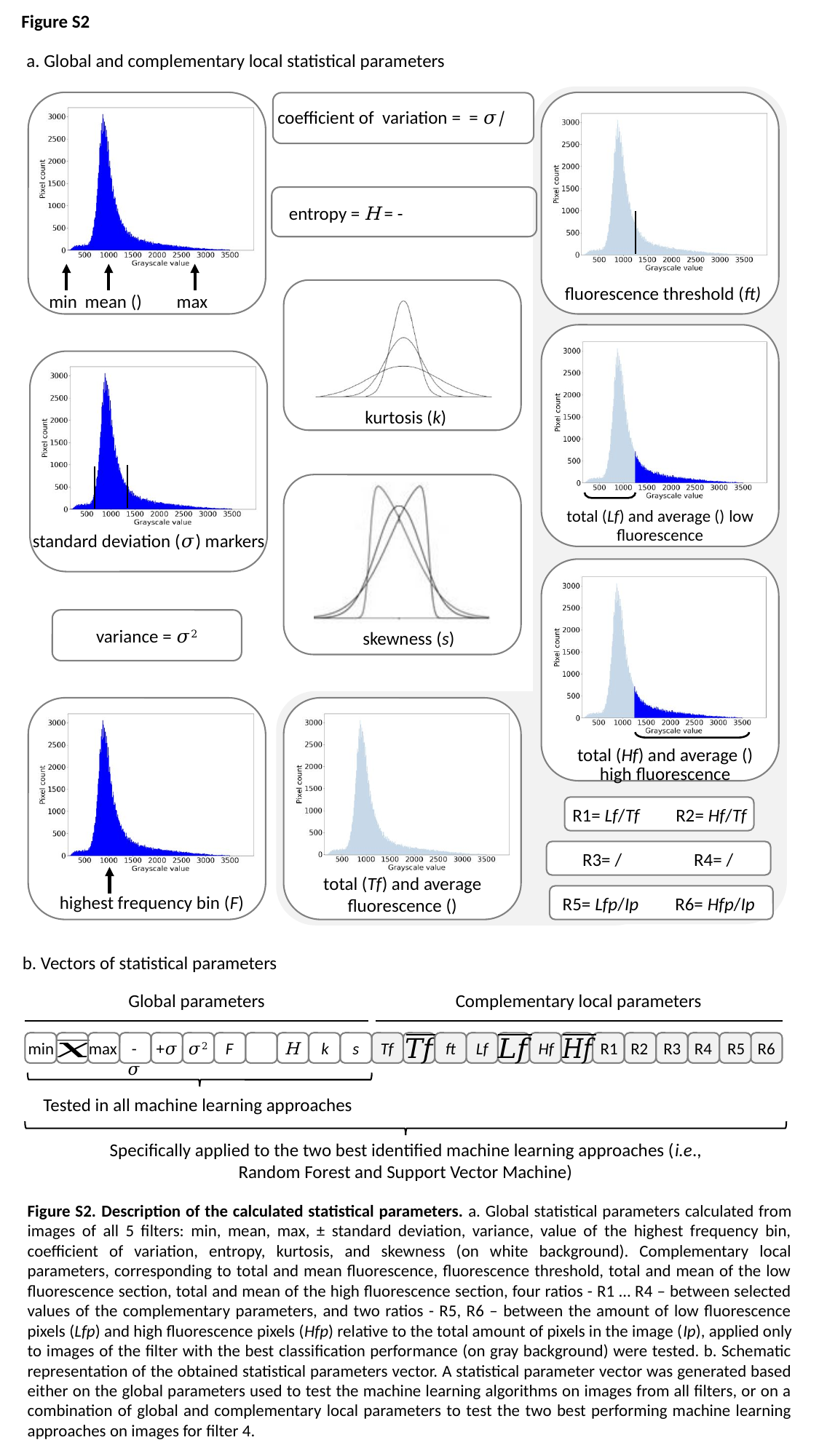

Figure S2
 a. Global and complementary local statistical parameters
fluorescence threshold (ft)
min
max
kurtosis (k)
standard deviation (𝜎) markers
skewness (s)
variance = 𝜎2
highest frequency bin (F)
R1= Lf/Tf
R2= Hf/Tf
R5= Lfp/Ip
R6= Hfp/Ip
b. Vectors of statistical parameters
Global parameters
Complementary local parameters
min
max
-𝜎
+𝜎
𝜎2
F
H
k
s
Tf
ft
Lf
Hf
R1
R2
R3
R4
R5
R6
Tested in all machine learning approaches
Specifically applied to the two best identified machine learning approaches (i.e., Random Forest and Support Vector Machine)
Figure S2. Description of the calculated statistical parameters. a. Global statistical parameters calculated from images of all 5 filters: min, mean, max, ± standard deviation, variance, value of the highest frequency bin, coefficient of variation, entropy, kurtosis, and skewness (on white background). Complementary local parameters, corresponding to total and mean fluorescence, fluorescence threshold, total and mean of the low fluorescence section, total and mean of the high fluorescence section, four ratios - R1 … R4 – between selected values of the complementary parameters, and two ratios - R5, R6 – between the amount of low fluorescence pixels (Lfp) and high fluorescence pixels (Hfp) relative to the total amount of pixels in the image (Ip), applied only to images of the filter with the best classification performance (on gray background) were tested. b. Schematic representation of the obtained statistical parameters vector. A statistical parameter vector was generated based either on the global parameters used to test the machine learning algorithms on images from all filters, or on a combination of global and complementary local parameters to test the two best performing machine learning approaches on images for filter 4.
